# Semen Quality and Freezability Analyses in the Ejaculates of Two Poitou Donkeys in the Southern Hemisphere

**DOI:** 10.3389/fvets.2021.662887

**Published:** 2021-09-03

**Authors:** Francisca Ebel, Omar Ulloa, Pablo Strobel, Alfredo Ramírez-Reveco

**Affiliations:** ^1^Instituto de Ciencia Animal, Facultad de Ciencias Veterinarias, Universidad Austral de Chile, Valdivia, Chile; ^2^Haras Militar Pupunahue, DGFER-Ejército de Chile, Los Lagos, Chile

**Keywords:** Baudet du Poitou, donkey, sperm quality, seminal plasma, cryopreservation, Southern hemisphere

## Abstract

The Baudet du Poitou is a vanishing donkey breed recognized for engendering robust working mules. In Chile, only two pure breed Poitou males exist, which belong to the Chilean army and are used for mule production. We performed an extensive sperm and seminal analysis of these two jackasses aged 3 and 6 years and investigated the use of a simple hypometabolic extender for sperm cryopreservation. Computer-assisted sperm analysis showed high motility, velocity, and linearity in sperm movement. The seminal plasma analysis revealed that sodium and chloride were the main electrolytes, and globulins were the main metabolites. Active and variable enzymatic activity was observed. New information is reported about gamma-glutamyltransferase, aspartate aminotransferase, zinc, and magnesium concentrations in seminal plasma of Poitou donkeys. Ejaculates among jackasses showed some variability due to individual variability and different stages in sexual maturation according to age. The freezability index analysis based in viability, total motility and progressive motility with Botucrio extender (57.1 ± 11.0%; 56.6 ± 20.0%; and 22.6 ± 10.3%, respectively) were significantly higher (*p* < 0.05, *p* < 0.0001, and *p* < 0.0001, respectively) than with HM-0 extender (42,6 ± 11.4%; 14.9 ± 5.1%; and 1.0 ± 2.5%, respectively). We report new information on Poitou donkey semen and cryopreservation in the Southern Hemisphere that could be useful in donkey breeding and conservation programs to develop strategies that improve the effectiveness of population management of this breed.

## Introduction

Donkeys are an important socio-economic support for human livelihood (1, 2). One of the oldest domestic donkey breeds is the Baudet du Poitou (Studbook since 1884). This French breed is highly recognized and requested for engendering robust mules useful in agricultural and army activities (3). This breed is classified as “endangered-maintained,” which indicates a worldwide population of pure breed animals of up to 6–20 males and 100–1,000 females (4). The steady decrease in the population is due to the industrialization of agriculture and the closure of national stud farms in the 20th century (5). There is limited empirical information on the current world population of Poitou donkeys because they are mainly owned by hobby-breeders of small farms (6) and breeders' organizations (i.e., UPRA and SABAUD^1^), with a small number of animals per farm (4). This situation hinders the census logistics of their population by statistical organizations (7). Thus, information on the Poitou donkey population is mainly provided by breeders' associations or organizations through informal data (CREGENE, 2021^2^) According to recent data from the Domestic Animal Diversity Information System (DAD-IS) for 2018, there were 2,903 Baudet du Poitou donkeys in the world−1,648 females and 486 males. Ninety-nine females of this population are pure breed and only five males participate in the national cryopreservation bank for insemination programs (4). Besides, in France, which has the greatest population of this breed, the French Institute of Horse and Riding reported 117 new births in 2017 (IFCE, 2019)^3^ Nonetheless, the Rare Breeds Trust of Australia, which declared two animals of this breed, reported that the international status of the Poitou donkeys is “critical” and estimated that there are fewer than 200 Poitou Donkeys in the world today (RBTA, 2019^4^) Similarly, in the United States, the University of Illinois Veterinary Teaching Hospital, which keeps five Poitou jennies, stated that fewer than 70 and 500 Poitou donkeys remain throughout the United States and the world, respectively (University of Illinois College of Veterinary Medicine, 2020^e^). In South America, only five Poitou donkeys were reported (three females and two males). These specimens belong to the Chilean army and are used to produce mules, whose purpose is the transport of supplies and materials to mountain areas, which are otherwise difficult to reach (8).

To preserve the livestock genetic diversity and populations of Poitou donkeys, it is crucial to know their normal seminal characteristics and develop efficient techniques for semen cryopreservation that will ensure optimal results in conservation programs. Consequently, several studies have reported the importance of seminal plasma for spermatozoa (9–14), semen preservation (15, 16), and reproduction (17, 18). Donkeys differ strikingly in their reproductive process from horses and among donkey breeds, which is why data comparisons between donkeys and horses and between donkeys of different breeds are questionable (9, 12). Research on fresh and cryopreserved semen of Poitou donkeys has been performed (9, 18–24) but a detailed study of semen characterization, including biochemical analysis of semen, has been only partially conducted in this breed.

All investigations on fresh and cryopreserved Poitou ejaculates have been performed only in the Northern Hemisphere. Since fertility in related species such as stallions is higher in the Southern than in the Northern Hemisphere (25), we fulfill the requirements to analyze the ejaculates' behavior of Poitou jackass in the Southern Hemisphere and compare it with that of Northern Hemisphere jackasses. This study aimed (1) to perform an extensive semen analysis, including sperm quality and biochemical profile of seminal plasma, on the ejaculates of the two Poitou jackasses aged 3 and 6 years; and (2) to investigate the effect of a simple hypometabolic extender on sperm cryopreservation.

## Materials and Methods

### Animals and Semen Samples

Semen was obtained from two fertile Poitou jackasses belonging to the Chilean army (Haras Militar Pupunahue, Los Lagos, Chile; 39°47′32″S, 72°53′34 ″O). Jackass 1 was 6 years old and weighed 450 kg. Jackass 2 was 3 years old and weighed 380 kg. Both animals were fed in *ad libitum* pasture and water conditions. As these donkeys are used in labor activities, alfalfa hay and corn supplementation were also provided twice daily. Animals were kept in paddocks and only if the weather conditions were adverse during the winter were they housed in a barn.

Donkey semen without extra-gonadal sperm reserves (with Daily Sperm Output [DSO] stabilized) was collected using an artificial vagina of an estrus female. Fourteen ejaculates (seven from each jackass) were collected during the breeding season (summer). All ejaculates were used for sperm evaluation, and 12 (6 from each donkey) were used for biochemical evaluation.

### Semen Evaluation

#### Sperm Quality

Seminal volume was determined by graduated containers, removing the gel-free fraction, and sperm concentration was estimated with a spectrophotometer (AccuRead, IMV Technologies, France).

For the sperm analysis, 40 μl of each ejaculate was diluted 1:5 in UHT skim milk and kept at 37°C for motility assessment. The remaining sample was diluted 1:1 with skim milk and kept at 37°C for concentration, viability, and morphology evaluation.

Plasma membrane integrity (viability) and morphology were assessed by using an eosin-nigrosin staining dye technique (18, 19, 26). Briefly, the sperm samples were mixed 1:1 with a stain solution (50 g/L eosin; 100 g/L nigrosin) on a tempered microscope slide and analyzed under a bright field microscope (Nikon Eclipse E200). The percentage of sperm viability was obtained according to the permeability of the plasma membrane of the sperm head to the eosin. The sperm morphology was determined as the percentage of normal spermatozoa without morphological abnormalities in the head, midpiece, and tail. At least 500 spermatozoa for each sample were analyzed in triplicate.

Total and progressive motility and sperm kinetic parameters were assessed using computer-assisted sperm analysis (CASA system, SCA, Microptic S.L., Spain) according to Córdova et al. (27). Aliquots of the sperm samples were then briefly placed on a pre-warmed slide at 37°C. The samples were analyzed by using a phase-contrast microscope (Nikon Eclipse E200) with 10× magnification (negative phase), coupled with a high-velocity camera (scA780 54tc). Twenty-five consecutive photographs were taken per second and at least 500 spermatozoa for each sample were analyzed in triplicate in three to six separate fields for each sample. For sperm kinetic analysis, the following parameters were considered: curvilinear velocity (VCL), linear velocity (VSL), mean velocity (VAP), linearity coefficient (LIN), straightness coefficient (STR), wobble coefficient (WOB), mean amplitude of lateral head displacement (ALH), and frequency of head displacement (BCF). Following these parameters, total motility was defined as the percentage of spermatozoa showing a VCL of above 10 μm/s, and progressive motility was defined as the percentage of spermatozoa showing an STR above 75%. At least six fields and 500 spermatozoa were measured in each evaluation.

#### Biochemical Evaluation

Fifteen milliliters of each ejaculate (gel-free fraction) was centrifuged twice at 2,700×g for 15 min. The supernatant of each tube was stored at −20°C until pH [PL-600] and osmolarity (Osmomat030) analyses. The enzymes, ions, and metabolites of the seminal plasma were analyzed by Laboratorio de Patología Clínica of Universidad Austral de Chile (Valdivia, Chile). The following parameters were considered in the analysis: (a) enzymes: alkaline phosphatase [[ALP]; alkaline phosphatase liquicolor], aspartate aminotransferase [[AST]; GOT IFCC mod. LiquiUV], and gamma-glutamyltransferase [[GGT]; γ-GT liquicolor]; (b) ions: total calcium [[Ca2+]; calcium liquicolor], inorganic phosphorus [[Pi]; phosphorus liquirapid], magnesium [[Mg2+]; magnesium liquicolor], sodium [[Na+]; atomic absorption spectrophotometry], chloride [[Cl–]; chloride liquicolor], potassium [[K+]; potassium liquirapid], zinc [[Zn2+]; atomic absorption spectrophotometry], and Ca:Pi ratio; (c) metabolites: cholesterol (cholesterol liquicolor), total protein (total protein liquicolor), albumin (albumin liquicolor), globulins (total protein minus albumin), urea (urea liquiUV), and glucose (glucose liquicolor). All tests were supplied by HUMAN (Weisbaden, Germany).

## Freezability Test

### Freezing Protocol

Fresh semen samples with >70% viability and total motility were diluted in UHT skim milk at 37°C, centrifuged, and resuspended in the following freezing extenders (80 ×106 sperm/ml):

- The commercial extender Botucrio (Botupharma, Brazil). Osmolarity: 1,244 ± 1 mOsm/L. pH: 7.4. Glucose concentration: 99.0 ± 6.1 mM (mean ± SD).
- The hypometabolic extender HM-0 Tris, composed of 300 mM Tris base, 94.7 mM citric acid, 2% (v:v) glycerol, 15% (v:v) egg yolk, 0.5 mg/ml streptomycin sulfate, and 0.05 mg/ml gentamicin. Osmolarity: 592 ± 4 mOsm/L. pH: 7.4. Glucose concentration: 1.9 ± 0.1 mM (mean ± SD) (27).

The freezing–thawing protocol and the freezing extender used were based on Cordova et al. (27). Briefly, ejaculates were collected using an artificial vagina, filtered to remove the gel, diluted 1:1 in pre-warmed UHT skimmed milk (isothermal condition), and centrifuged to 1,000×*g* for 20 min. Post-centrifugation sperm pellets were suspended in the extenders previously tempered at 20°C, packed in 0.5 ml at 50 × 10^6^ sperm/ml in straws, and cooled to 5°C for 90 min. Afterward, straws were subsequently exposed to liquid nitrogen vapors for 20 min and finally plunged and stored in liquid nitrogen for at least 2 months before analysis. The temperature of the samples was recorded during the whole process using a temperature probe coupled to a USB data logger (ThermoWorks, Alpine-UT, USA).

### Post-thawed Sperm Evaluation

Samples were centrifuged and resuspended in Tris/citrate buffer extender for sperm quality evaluation. Each evaluation was established from a count of at least six different fields and 500 spermatozoa in each assessment.

The plasma membrane integrity (viability) was evaluated by the CASA system using Ethidium Bromide (EB)/Acridine Orange (OA) double staining technique according to Córdova et al. (27). Briefly, sperm samples were mixed (1:1) on a tempered microscope slide with a staining aqueous solution composed of 10 μM EB and 20 μM AO (EB/AO solution). Samples were immediately visualized and analyzed using the viability module from the CASA system (Sperm Class Analyzer, Microptic, Spain) coupled to an epifluorescence microscope (Nikon E200, upright microscope) with a high-velocity camera (Basler AG, Germany, scA780 54tc). Viable spermatozoa were green-stained on the head, whereas non-viable spermatozoa were red-stained on the head. The sperm motility was evaluated by the CASA system as previously described for fresh samples.

The acrosome integrity analysis was evaluated by FITC-PSA dye (Sigma Aldrich, USA), according to Ramirez et al. (28). Briefly, sperm aliquots were fixed and permeabilized for at least 30 min at 4°C in 100% methanol, to allow entry of PSA. Permeabilized spermatozoa, dried onto slides, were then covered with a droplet of 100 mg/ml FITC-PSA PBS for 10 min. Later, the slides were washed with bi-distilled water, and the spermatozoa were analyzed with an inverted epifluorescence microscope (Leica DMI3000 B). The emission fluorescence of PSA-FITC was detected using a 450- to 490-nm UV excitation filter, a 510-nm dichroic mirror, and a 520-nm barrier filter. Data were analyzed in triplicate. Intense acrosome staining was indicative of an intact acrosome. Sperm with structurally altered acrosomes were those displaying a slight fluorescence or no fluorescence at all on the sperm head. To compare the extender freezing ability, a Freezability Index (FI) was calculated for viability and total and progressive motility [FI [%] = AF/BF × 100; sperm values after [VAF] and values before freezing [VBF]].

## Statistical Analysis

Data were analyzed using GraphPad Prism 6 statistical software (USA) and they included mean values, standard deviation (mean ± SD). For comparative analysis of post-thawing semen parameters and freezability analysis between freezing extenders, the paired sample *t*-test were used. *p*-values of <0.05 and <0.01 were considered significant and highly significant differences, respectively.

## Results

### Semen Evaluation

#### Sperm Quality

The results of sperm quality and motility parameters of both Poitou donkeys are shown in Tables 1, 2, respectively. The mean values for jackass 1 and 2 were 52.9 ± 18.0 and 44.3 ± 14.0 ml of free-gel fraction volume, 552.9 ± 122.8 and 198.6 ± 55.6 × 106/ml of sperm concentration, 77.8 ± 5.5 and 85.6 ± 5.3% of viability, and 74.7 ± 6.9 and 79.9 ± 9.9% of normal morphology, respectively. Concerning the overall means, free-gel fraction volume, viability, and normal morphology showed slight dispersion with values of 48.6 ± 16.1 ml, 81.7 ± 6.6%, and 77.3 ± 8.7%, respectively. In contrast, the overall mean of sperm concentration showed a high dispersion with a value of 375.7 ± 205.4 × 106/ml.

**Table 1:** Free-gel fraction volume, sperm concentration, viability, and normal morphology of fresh Poitou jackass ejaculates.

**Parameters**	**Jackass 1**	**Jackass 2**	**Range**		**Trimeche et al**.	**Gupta et al**.	**Talluri et al**.	**Kumar**	**Kumar et al**.	**Kumar et al**.
	**(J1)**	**(J2)**	**(J1** **+** **J2)**		**(22)**	**(18)**	**(9)**	**(29)**	**(19)**	**(30)**
	**Mean ± SD**	**Mean ± SD**	**Mean ± SD**	**95% Confidence interval**	**Mean ± SD**	**Mean ± SD** ^**a**^	**Mean ± SD** ^**a**^	**Mean ± SD** ^**a**^	**Mean ± SD**	**Mean ± SD** ^**a**^
Free-gel fraction volume (ml)	52.9 ± 18.0	44.3 ± 14.0	48.6 ± 16.1	40.2–58.0	-	43.7 ± 11.5	64.5 ± 7.1	45.9 ± 23.9	44.4 ± 3.9	47.0 ± 27.2
Sperm concentration (10^6^/ml)	552.9 ± 122.8	198.6 ± 55.6	375.7 ± 205.4	268.1–483.3	–	331.56 ± 67.3	262.3 ± 15.9	292.6 ± 22.7	282.1 ± 4.8	274.9 ± 5.6
Viability (%)	77.8 ± 5.5	85.6 ± 5.3	81.7 ± 6.6	78.3–85.1	73.6 ± 7.9	84.3 ± 3.5	85.2 ± 0.8	86.8 ± 9.4	91.8 ± 0.3	87 ± 2.0
Morphology (%)	74.7 ± 6.9	79.9 ± 9.9	77.3 ± 8.7	82.2–93.4	–	–	96.7 ± 0.85	91.9 ± 2.3	91.3 ± 0.26	91 ± 0.6
Number of donkeys	1	1	2		4	5	6	4	4	6
Total number of ejaculates	7	7	14		40	150	60	24	32	48
Donkey age (years)	3	6	3–6		–	–	–	4–6	–	4–7
Donkey weight (kg)	380	450	380–450		–	–	–	–	–	–

a *Values of standard error of the mean were transformed to standard deviation*.

**Table 2:** Motility and kinetic parameters of fresh Poitou jackass ejaculates.

**Parameters**	**Jackass 1 (J1)**	**Jackass 2 (J2)**	**Range**		**Trimeche et al**.	**Trimeche et al**.	**Gupta et al**.	**Talluri et al**.	**Kumar**	**Kumar et al**.	**Kumar et al**.
			**(J1** **+** **J2)**		**(21)**	**(20)**	**(18)**	**(9)**	**(29)**	**(19)**	**(30)**
	**Mean ± SD**	**Mean ± SD**	**Mean ± SD**	**95% Confidence interval**	**Mean ± SD** ^**a**^	**Mean ± SD** ^**a**^	**Mean ± SD** ^**a**^	**Mean ± SD** ^**a**^	**Mean ± SD** ^**a**^	**Mean ± SD** ^**a**^	**Mean ± SD** ^**a**^
Total motility (%)	90.1 ± 6.2	85.6 ± 8.6	87.8 ± 7.6	82.2–93.4	71.3 ± 8.4	70.3 ± 7.4	84.9 ± 6.4	–	–	–	84.0 ± 4.3
Progressive motility (%)	70.9 ± 8.8	56.4 ± 13.9	63.6 ± 13.5	56.5–70.7	39.1 ± 5.1	56.2 ± 5.1	75.1 ± 6.9	81.5 ± 0.9	80.1 ± 7.2	88.5 ± 0.4	80 ± 4.4
VCL (μm/s)	103.2 ± 5.4	91.0 ± 10.2	97.1 ± 10.1	89.6–104.6	60.6 ± 7.2	61.3 ± 6.8	–	–	–	–	–
VSL (μm/s)	66.3 ± 12.0	58.3 ± 13.8	62.3 ± 13.1	52.6–72.0	42.0 ± 4.2	45.2 ± 4.52	–	–	–	–	–
VAP (μm/s)	80.2 ± 10.4	72.9 ± 12.9	76.6 ± 11.9	67.8–85.4	38.3 ± 3.1	39.8 ± 3.4	–	–	–	–	–
STR (%)	82.3 ± 5.0	79.3 ± 6.2	80.8 ± 5.6	76.6–85.0	–	–	–	–	–	–	–
LIN (%)	65.0 ± 9.1	63.7 ± 11.1	64.3 ± 9.8	57.1–71.5	74.0 ± 8.7	74.6 ± 6.8	–	–	–	–	–
WOB (%)	78.7 ± 6.9	79.9 ± 8.1	79.3 ± 7.3	73.9–84.7	–	–	–	–	–	–	–
ALH (μm)	3.6 ± 0.4	3.1 ± 0.7	3.3 ± 0.6	2.8–3.8	3.2 ± 0.4	4.1 ± 0.6	–	–	–	–	–
BCF (Hz)	10.1 ± 0.9	8.8 ± 0.7	9.5 ± 1.0	8.8–10.2	13.9 ± 1.9	–	–	–	–	–	–
Number of donkeys	1	1	2		4	4	5	6	4	4	6
Number of ejaculates	7	7	14		40	32	150	60	24	32	48
Donkey age (years)	3	6	3–6		–	3–7	–	–	4–6	-	4–7
Donkey weight (kg)	380	450	380–450		–	300–500	–	–	–	–	–

a *Values of standard error of the mean were transformed to standard deviation*.

*VCL, curvilinear velocity; VSL, linear velocity; VAP, mean velocity; LIN, linearity coefficient; STR, straightness coefficient; WOB, wobble coefficient; ALH, mean amplitude of lateral head displacement; BCF, frequency of head displacement*.

Regarding motility parameters, the mean values for both jackass 1 and 2 were 90.1 ± 6.2 and 85.6 ± 8.6% of total motility and 70.9 ± 8.8 and 56.4 ± 13.9% of progressive motility, respectively, with total and progressive motility overall means of 87.8 ± 7.6 and 63.6 ± 13.5%, respectively. The kinetic evaluation revealed that the sperm of both donkeys exhibited high velocity (VCL: 97.1 ± 10.1, VSL: 62.3 ± 13.1, and VAP 76.6 ± 11.9 μm/s) and linearity (STR: 80.8 ± 5.6, LIN: 64.3 ± 9.8, and WOB: 79.3 ± 7.3%) in their sperm movement.

#### Biochemical Evaluation

The overall seminal plasma values of pH and osmolarity were 7.42 ± 0.1 and 285.7 ± 6.5 mOsm/kg, respectively. The enzymatic evaluation revealed the presence of active and variable ALP, AST, and GGT activity in the seminal plasma of both Poitou donkeys. Thus, mean values and range for ALP, AST, and GGT were 338.0 ± 81.5 and 206.9 ± 56.5 [231.9 to 362.9], 4.9 ± 0.1 and 3.4 ± 1.2 [3.4 to 4.9], and 301.8 ± 215.5 and 272.0 ± 146.5 [187.1 to 386.7] μkat/L for jackass 1 and 2, respectively (Table 3).

**Table 3:** pH, osmolarity, and enzyme profile of Poitou jackass seminal plasma.

**Parameters**	**Jackass 1** **(J1)**	**Jackass 2** **(J2)**	**Range (J1** **+** **J2)**		**Gupta et al. (18)**	**Kumar (29)**	**Talluri et al. (9)**	**Kumar et al. (30)**
	**Mean ± SD**	**Mean ± SD**	**Mean ± SD**	**95% Confidence interval**	**Mean ± SD** ^**a**^	**Mean ± SD** ^**a**^	**Mean ± SD** ^**a**^	**Mean ± SD** ^**a**^
pH	7.40 ± 0.1	7.45 ± 0.1	7.42 ± 0.1	7.42–7.42	7.31 ± 0.3	7.73 ± 0.2	7.30 ± 0.0	7.45 ± 0.4
Osmolarity (mOsm/kg)	285.7 ± 4.2	285.7 ± 8.7	285.7 ± 6.5	282.0–289.4	–	–	–	–
***Enzymes***								
ALP (μkat/L)	338.0 ± 81.5	206.9 ± 56.5	297.5 ± 115.8	231.9–362.9	0.9 ± 0.2	-	0.2 ± 0.08	–
AST (μkat/L)	4.9 ± 0.1	3.4 ± 1.2	4.1 ± 1.3	3.4–4.9	–	–	–	–
GGT (μkat/L)	301.8 ± 215.5	272.0 ± 146.5	286.9 ± 176.4	187.1–386.7	–	–	–	–
Number of donkeys	1	1	2		5	4	6	6
Number of ejaculates	6	6	12		150	24	60	48
Donkey age (years)	3	6	3–6		–	4–6	–	4–7
Donkey weight (kg)	380	450	380–450		–	–	–	–

a *Values of standard error of the mean were transformed to standard deviation*.

*ALP, alkaline phosphatase; AST, aspartate aminotransferase; GGT, gamma-glutamyltransferase*.

Regarding ion concentrations in seminal plasma (Table 4), sodium and chloride were the main electrolytes in seminal plasma in both donkeys with overall values of 130.0 ± 21.3 and 128.5 ± 9.3 mmol/L, respectively. Total calcium and inorganic phosphorus overall concentrations were 1.8 ± 0.5 and 1.4 ± 0.6 mmol/L, respectively, with a Ca:Pi ratio of 1.3 ± 0.2. The concentrations of potassium and magnesium for both jackasses were 11.0 ± 1.6 and 2.2 ± 1.1 mmol/L, respectively. Low concentrations of zinc were also observed with a value of 20.9 ± 5.0 μmol/L.

**Table 4:** Ion profile of Poitou jackass seminal plasma.

**Parameters**	**Jackass 1** **(J1)**	**Jackass 2** **(J2)**	**Range (J1** **+** **J2)**		**Gupta et al. (18)**	**Talluri et al. (9)**
	**Mean ± SD**	**Mean ± SD**	**Mean ± SD**	**95% Confidence interval**	**Mean ± SD** ^**a**^	**Mean ± SD** ^**a**^
Total calcium (mmol/L)	2.1 ± 0.6	1.5 ± 0.1	1.8 ± 0.5	1.5–2.1	–	4.5 ± 2.2
Inorganic phosphorus (mmol/L)	1.8 ± 0.6	1.1 ± 0.2	1.4 ± 0.6	1.1–1.7	–	3.8 ± 1.5
Ca:Pi ratio	1.2 ± 0.2	1.4 ± 0.3	1.3 ± 0.2	1.2–1.4	–	–
Magnesium (mmol/L)	2.6 ± 1.5	1.8 ± 0.3	2.2 ± 1.1	1.6–2.8	–	–
Sodium (mmol/L)	116.2 ± 4.3	143.8 ± 22.8	130.0 ± 21.3	118.0–142.0	110.8 ± 5.9	-
Chloride (mmol/L)	121.5 ± 3.0	135.5 ± 8.1	128.5 ± 9.3	123.2–133.8	115.2 ± 5.6	–
Potassium (mmol/L)	12.0 ± 1.5	10.1 ± 1.0	11.0 ± 1.6	18.1–23.7	19.7 ± 0.9	–
Zinc (μmol/L)	23.5 ± 5.8	18.3 ± 2.5	20.9 ± 5.0	18.1–23.7	–	–
Number of donkeys	1	1	2		5	6
Number of ejaculates	6	6	12		150	60
Donkey age (years)	3	6	3–6		–	–
Donkey weight (kg)	380	450	380–450		–	–

a *Values of standard error of the mean were transformed to standard deviation*.

As shown in Table 5, the analysis of seminal plasma metabolites revealed that globulins were the main protein of seminal plasma in these jackasses (27.3 ± 6.3 g/L and 28.0 ± 6.5 g/L of total protein). Additionally, low concentrations of cholesterol and glucose in seminal plasma were found in both donkeys, with values of 0.2 ± 0.1 and 0.1 ± 0.1 mmol/L, respectively. The urea concentration in ejaculates of these jackasses was 6.6 ± 0.7 mmol/L.

**Table 5:** Metabolite profile of Poitou jackass seminal plasma.

**Parameters**	**Jackass 1** **(J1)**	**Jackass 2** **(J2)**	**Range (J1** **+** **J2)**		**Gupta et al. (18)**	**Talluri et al. (9)**
	**Mean ± SD**	**Mean ± SD**	**Mean ± SD**	**95% Confidence interval**	**Mean ± SD** ^**a**^	**Mean ± SD** ^**a**^
Cholesterol (mmol/L)	0.2 ± 0.1	0.2 ± 0.1	0.2 ± 0.1	0.1–0.3	16.3 ± 5.6	0.6 ± 0.2
Total protein (g/L)	32.7 ± 4.7	23.3 ± 4.3	28.0 ± 6.5	24.3–31.7	50.4 ± 6.0	40.3 ± 9.0
Globulins (g/L)	32.0 ± 3.9	23.3 ± 4.3	27.3 ± 6.3	23.8–30.8	28.1 ± 4.0	–
Urea (mmol/L)	6.2 ± 0.3	7.0 ± 0.7	6.6 ± 0.7	6.2–7.0	6.2 ± 4.1	–
Glucose (mmol/L)	0.1 ± 0.1	0.1 ± 0.1	0.1 ± 0.1	0.0–0.2	0.5 ± 0.2	1.4 ± 0.6
Number of donkeys	1	1	2		5	6
Number of ejaculates	12	12	12		150	60
Donkey age (years)	3	6	3–6		–	–
Donkey weight (kg)	380	450	380–450		–	–

a *Values of standard error of the mean were transformed to standard deviation*.

## Post-thawing Sperm Quality and Freezability Analysis

**Figure 1:**
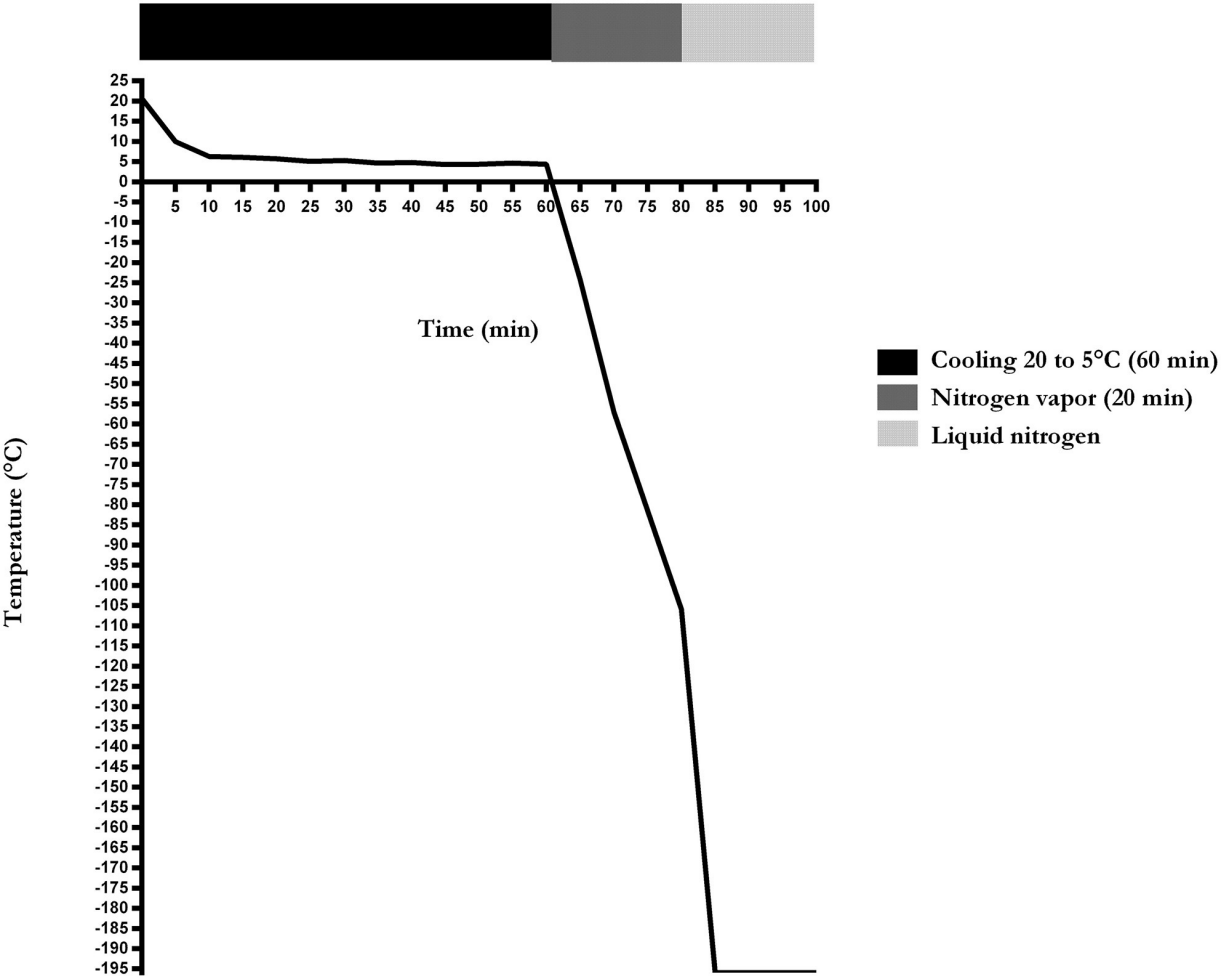
Cooling and freezing rate of sperm cryopreservation of Poitou jackasses (*n* = 14).

The cooling and freezing rates of the cryopreservation protocol were −0.3° and −5.5°C/min, respectively. Sperm achieved −105.9°C before being immersed in liquid nitrogen (Figure 1).

**Figure 2:**
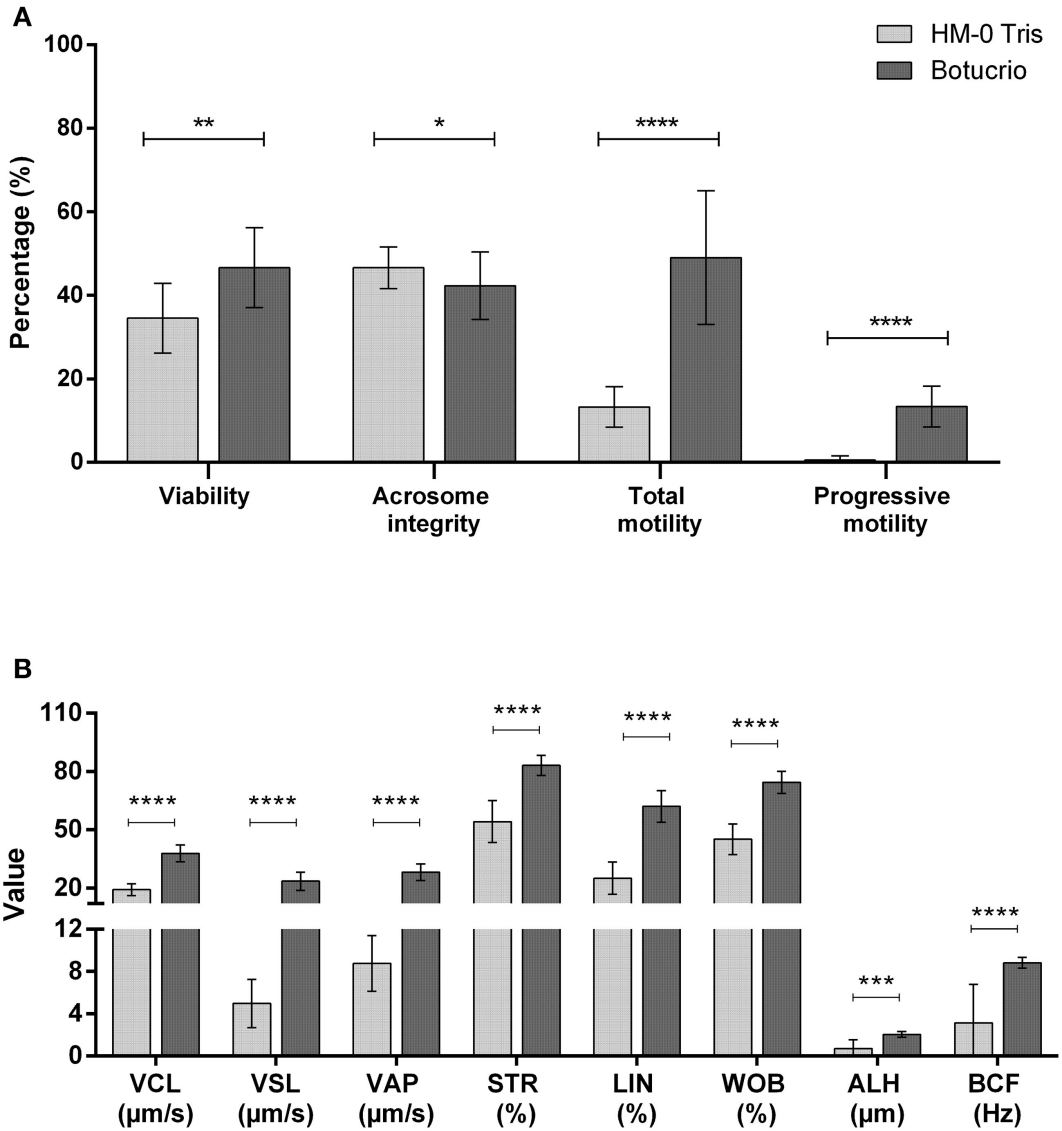
Post-thawed results of Poitou jackass sperm cryopreserved with HM-0 Tris and Botucrio extenders. **(A)** Viability, acrosome integrity, and total and progressive motility. **(B)** Kinetic parameters. VCL, curvilinear velocity; VSL, linear velocity; VAP, mean velocity; LIN, linearity coefficient; STR, straightness coefficient; WOB, wobble coefficient; ALH, mean amplitude of lateral head displacement; BCF, frequency of head displacement. Each bar represents mean ± SD (*n* = 14). Paired *t*-test analysis; significant differences between two extenders (^*^p < 0.05; ^**^p < 0.01; ^***^p < 0.001; ^****^p < 0.0001) are shown.

The comparative results are shown in Figure 2, in which post-thawed sperm viability, total motility, and progressive motility, as well as all kinetic parameters (except for ALH) were significantly higher when using the Botucrio extender than the HM-0 extender (*p* < 0.01). The only one parameters in which the result using Botucrio extender was significantly lower than with the HM-0 extender was acrosome integrity (*p* < 0.05).

**Figure 3:**
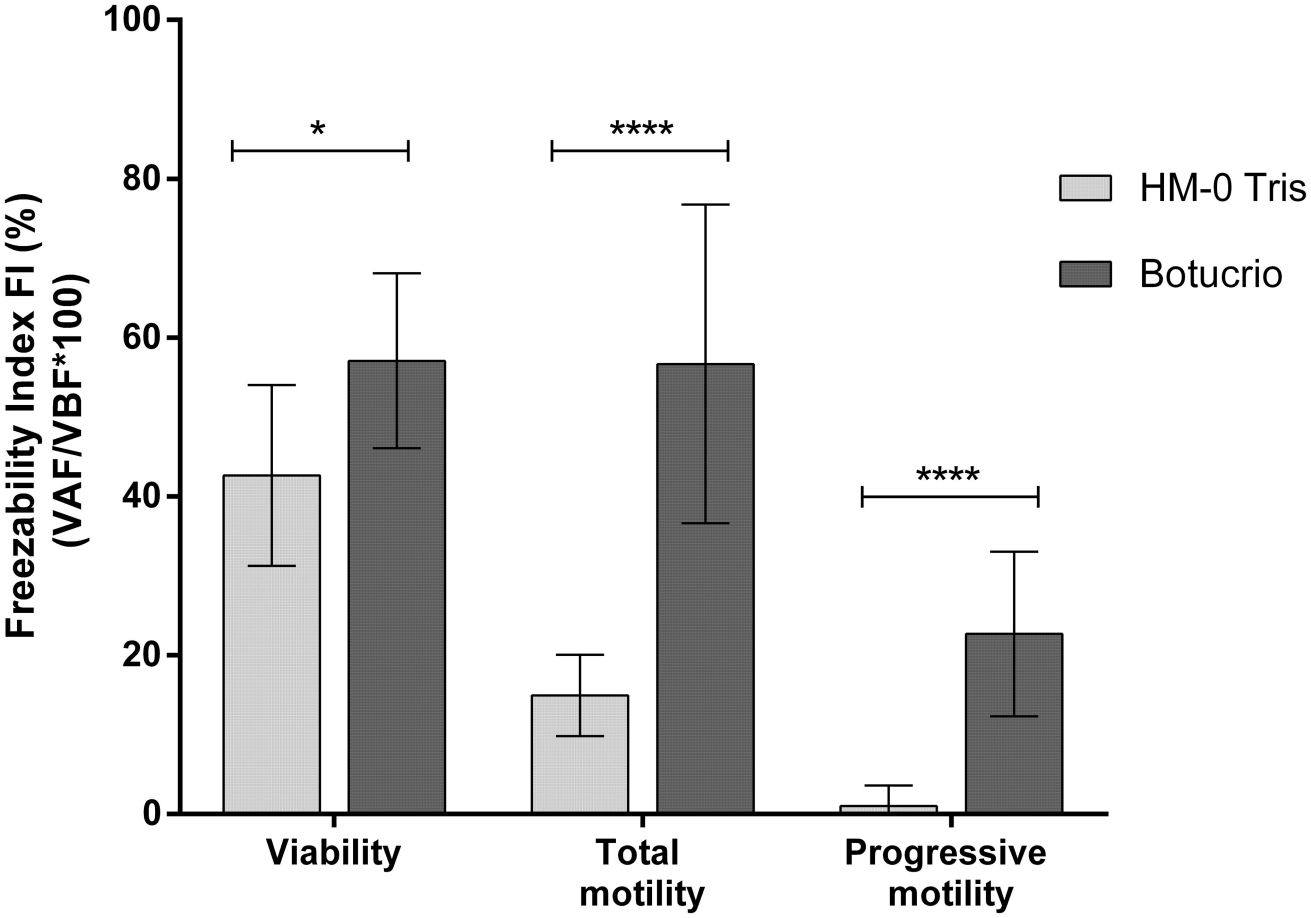
Freezability index (FI) of viability and total motility in Poitou jackass sperm cryopreserved with Botucrio and HM-0 Tris extenders. Each bar represents mean ± SD (*n* = 14). Paired *t*-test analysis; significant differences between two extenders (^*^p < 0.05 and ^****^p < 0.0001) are shown.

Finally, the freezability analysis (Figure 3) has shown that FI of the three parameters considered were significantly higher when the Botucrio extender rather than the HM-0 extender was used (viability: *p* < 0.05; total and progressive motility: *p* < 0.0001).

## Discussion

The results of the sperm quality analysis in the present study agree with those of other studies on Poitou jackasses performed in the Northern Hemisphere (9, 18–22, 30). These studies were done under similar conditions of animal number and age (26 animals and 4–7 years of age), but with higher repetitions than in the present study (24–150 ejaculates vs. 14 ejaculates). Despite these similarities, differences were observed in normal morphology and progressive motility values between our study and those mentioned above. The lower normal morphology observed in our study (77.3 vs. >90%) could be due to the criterion used to detect structural abnormalities despite the fact that other authors used the same dye technique (9, 18, 19). The progressive motility reported in our study (63.6%) was higher than that observed by Trimeche et al. (20, 21) (39–56%), but lower than that of the other studies (9, 18, 19, 30), which obtained progressive motility values over 75%. These dissimilarities may be related to differences in the extender composition (21), evaluation objectivity (9, 18), and CASA system configuration (9, 20, 30) used in each study. In addition to these results, this study reported new insights into the kinetic characteristics of Poitou donkey sperm. Thus, the sperm movement of these animals by CASA system showed high velocity and linearity indicators, with values of STR and LIN over 80 and 60%, respectively.

Enzymes, metabolites, and ions of seminal plasma are essential for sperm structure, metabolism, and fertilization. To the best of our knowledge, little information is available about the characterization of seminal parameters of Poitou donkeys. Here, we report a detailed analysis of seminal plasma of the only two pure Poitou sires existing in Chile. This analysis included the evaluation of pH, osmolarity, three enzymes, seven ions, and five metabolites of Poitou donkeys aged 3 and 6 years.

The biochemical profile of ions and metabolites in the seminal plasma of the donkeys analyzed here is similar to that reported for this breed in the Northern Hemisphere, where 60–150 ejaculates of 5–6 Poitou jackasses were analyzed (9, 18). However, the results of total protein, total calcium, inorganic phosphorus, and potassium of seminal samples in this study were lower than those reported by these authors. These differences may be related to the geographic location and nutrition of the donkeys among the studies. It is well-known that donkeys have lower maintenance energy requirements than other equids. Donkeys come from semi-arid and mountainous environments with sparse food sources and their digestive tract has adapted to a high-fiber and low-energy diet (31). The cited authors conducted their experiments in India, but they did not specify the exact latitude localization or the nutrition protocol of the studied donkeys. The present study was done at latitude 39°47′32″S, 72°53′34″O in southern Chile, and the donkeys were fed mainly natural pasture and supplemented with alfalfa hay and corn grain twice daily. The soil in southern Chile has a volcanic composition (32), and heavy rainfall during the winter months reduces soil nutrients, such as calcium, phosphorus, and magnesium, which could be implicated in the lower seminal concentrations of these minerals (33, 34). Little investigation has been done to establish the mineral requirements of donkeys (31). Further studies are needed to determine whether the composition of southern Chilean soil affects donkey seminal quality since these animals have been reported to have lower mineral requirements than other equids (35).

Sodium (118.0–142.0 mmol/L) and chloride (123.3–133.8 mmol/L) were the main ions, and globulins (23.8–30.8 g/L) were the main metabolites in the seminal plasma of the donkeys. Also, low concentrations of glucose (0.0–0.2 mmol/L), cholesterol (0.1–0.3 mmol/L), zinc (18.1–23.7 μmol/L), and magnesium (1.6–2.8 mmol/L) were found. Zinc is a micromineral that plays an important role in many protective sperm properties, such as membrane stabilization, and antioxidant and antibacterial functions (36), and a testicular and epididymal origin has been suggested in Equidae (11, 37). Magnesium is a cation related to high seminal plasma quality in boars because it improves viability by reducing sperm membrane damage (38). A prostatic origin of magnesium has also been described in humans (39). Interestingly, to date, no studies have reported zinc and magnesium levels in the seminal plasma of Poitou jackasses. Thus, the present study reports the seminal concentrations of zinc and magnesium in this breed, where the zinc level was similar and the magnesium level was higher than those found in other domestic donkeys by Vyvial et al. (37).

There is little information about the enzymatic activity of seminal plasma in donkeys. The GGT is an important antioxidant in semen that protects sperm against oxidative damage. This enzyme has been correlated with high sperm viability and motility in fresh samples from stallions (11). In bull, seminal GGT has been correlated with high sperm motility, embryo cleavage, and blastocyst rate with post-thawed sperm (40). In humans, low seminal concentrations of seminal GGT have been related to infertility (41). The AST and ALP have been related to membrane sperm integrity in humans (42). In donkeys, AST and ALP are secreted from testis and epididymis, while GGT's origin is not clear because it does not correlate with volume or sperm concentration (37). In stallion, GGT has a testicular and epididymal origin, but since it is species-specific, this information may not apply to jackass (11). The results of our study revealed that the seminal plasma of both donkeys had active and variable ALP, AST, and GGT enzymatic levels, with ALP and GGT showing the highest and most variable activity, in agreement with observations by Vyvial et al. (37) in seminal plasma of other domestic donkeys. In humans, these enzymes were reported to be significantly higher in seminal plasma than in serum, between 30 and 500 times higher (42), and these molecules might need to be highly concentrated in donkey seminal plasma, like other antioxidant enzymes, in order to protect the sperm (43). We have not found references of seminal AST or GGT concentrations for Poitou donkeys, but these values are apparently normal since AST and GGT levels (AST: 3.4–4.9 μkat/L, GGT: 187.1–386.7 μkat/L) are within the range reported for other domestic donkeys [AST: 0.7–5.1 and GGT: 72.7–1,853 μkat/L; (37)]. The ALP concentration found in this study (231.9–362.9 μkat/L) was significantly higher than that previously reported for Poitou jackasses (9, 18). This difference may be related to the sensitivity of the technique used for ALP detection by the authors because the ALP values obtained in this study were between the normal range reported for domestic donkeys [23.4–542.4 μkat/L; (37)]. ALP values are related to sperm concentration (11, 18) and considered a marker of ejaculation in stallions (17). Therefore, the high activity of ALP observed in both jackasses could be associated with the high sperm concentration, especially that obtained from jackass 1 (552.9 ± 122.8 × 10^6^/ml). Further studies are necessary to determine the importance of these enzymes in the quality of fresh, cooled, and thawed donkey sperm samples and their relation to fertility.

Variations in semen quality between Poitou sires and within the same individual are expected (18). These differences might be influenced by individual variations or the animal age (44, 45). Male donkeys reach puberty between 19 and 20 months of age where sperm in ejaculates can be observed, although at this age they have not completed their full sexual maturation (46). Therefore, both jackasses evaluated in this study were post-pubertal (3 and 6 years old), but there may be differences in sexual maturation between them due to jackass age, as the 3-year-old animal had not reached full sexual maturation in comparison with the older animal. Nipken and Wrobler (47) observed in 5- to 6-year-old donkeys an increase in germ cell number per testis, spermiogenesis, tubular diameter, length and development, and epithelial efficiency relative to 3-year-old donkeys, and proposed that 6 years of age is the plateau of maximal testicular function. Further research in testicular size, testicular blood flow, and serum testosterone concentration is necessary to understand sexual differences among post-pubertal jackasses, as previously done in stallions (48, 49). The variability in sperm quality observed between the jackasses in this study was also noted by Kumar (29) in healthy and fertile donkeys of the same breed and age. This information could explain variations in sperm and seminal results between donkeys and could be considered normal since no differences between ejaculates or donkeys were observed in the post-thawed evaluation (data not shown; *p* > 0.05).

Animal genetic resources need to be preserved as part of sustainable management, especially in endangered breeds. Here, we show that Botucrio extender was superior to HM-0 extender in preserving donkey sperm characteristics based on the three freezability index obtained (p < 0.05). HM-0 is a simple Tris-based extender composed by glycerol (3%) and egg yolk (15%) maintained more than 40% of viability after the cryopreservation process. These percentage is 26% lower than the percentage obtained with Botucrio extender, which contains a complex composition of antioxidants and cryoprotectants, in addition to glycerol (1%) and egg yolk (5%) (50). Since the glycerol concentration in both extenders coincided to that reported for donkey semen cryopreservation (22), the sperm membrane protective effect of HM-0 Tris extender, could be influenced by high concentration of lecithin and the low density lipoproteins of the egg yolk (51), which improves the protection in the post-thawed sperm membrane in Poitou jacks (20) and in other donkey breeds (52, 53). As expected, post-thawed motility parameters and the FI of motility were significantly higher when using the Botucrio extender (*p* < 0.05). This extender contains amino acids, carbohydrates, and N-methylformamide, which improve sperm post-thawed motility (20, 22, 50, 54). Further studies are necessary to verify if changes in component concentrations (glycerol and egg yolk) and new additives (amides, carbohydrates and amino acids) on HM-0 formulation improves the freezability index (viability and sperm motility) of donkey sperm using a cryopreservation extender based on Tris-glycerol-egg yolk. Additionally, the freezability analysis revealed that Botucrio extender preserve post-thaw viability (*p* < 0.05) and total and progressive sperm motility (*p* < 0.0001) better than HM-0 Tris extender. This report provides new information to the knowledge of Poitou donkey sperm and cryopreservation in the Southern Hemisphere, which may help donkey breeding and conservation programs to develop strategies to improve the effectiveness of population management for this breed.

## Data Availability Statement

The original contributions presented in the study are included in the article, further inquiries can be directed to the corresponding author/s.

## Ethics Statement

The animal study was reviewed and approved by Bioethics of Animals for Research (C#151-2014; UACh. Chile) and the Animal Welfare Law-Conicyt (Chile). Written informed consent and protocol of animal manipulation were obtained from Haras Militar Pupunahue (Chilean Army) for the participation of their animals in this study.

## Author Contributions

FE, OU, and AR-R contributed to conception and design of the study. FE, OU, and PS supported the assembly and development of methods and protocols for sampling and analysis of seminal quality parameters. FE organized the database, performed the statistical analysis, and wrote the first draft of the manuscript. AR-R contributed with formal analysis, funding acquisition, supervision, writing, review, and editing of final manuscript. All authors approved the submitted version.

## Conflict of Interest

The authors declare that the research was conducted in the absence of any commercial or financial relationships that could be construed as a potential conflict of interest.

## Publisher's Note

All claims expressed in this article are solely those of the authors and do not necessarily represent those of their affiliated organizations, or those of the publisher, the editors and the reviewers. Any product that may be evaluated in this article, or claim that may be made by its manufacturer, is not guaranteed or endorsed by the publisher.

## Footnotes

^1^ http://www.racesmulassieresdupoitou.com

^2^ http://www.cregene.org/Mamiferes.html

^3^ https://www.ifce.fr/ifce/connaissances/statistiques-et-donnees/

^4^ https://rarebreedstrust.com.au/
